# Different Influences on Tacrolimus Pharmacokinetics by Coadministrations of Zhi Ke and Zhi Shi in Rats

**DOI:** 10.1155/2011/751671

**Published:** 2011-01-20

**Authors:** Shiuan-Pey Lin, Ping-Ping Wu, Yu-Chi Hou, Shang-Yuan Tsai, Meng-Ju Wang, Shih-Hua Fang, Pei-Dawn Lee Chao

**Affiliations:** ^1^School of Pharmacy, China Medical University, Taichung 40402, Taiwan; ^2^School of Chinese Pharmaceutical Sciences and Chinese Medicine Resources, China Medical University, Taichung 40402, Taiwan; ^3^Institute of Athletics, National Taiwan College of Physical Education, Taichung 40404, Taiwan

## Abstract

Tacrolimus, an immunosuppressant with narrow therapeutic window, has been used widely in transplant patients. Grapefruit juice and pomelo have been reported to increase the blood levels of tacrolimus. Zhi Ke and Zhi Shi, the ripe peels and unripe fruits of *Citrus aurantium* which is chemotaxonomically related to grapefruit and pomelo, are in wide use in clinical Chinese medicine. To investigate the possible interaction of these two *Citrus* herbs with tacrolimus, male Sprague-Dawley rats were orally given tacrolimus (1.5 mg/kg) with and without Zhi Ke and Zhi Shi decoctions in a cross-over design. Blood samples were withdrawn via cardiopuncture at specific time and quantitated by a microparticle enzyme immunoassay. In addition, to explore the mechanism of interaction, LS 180 cell line was used for the transport study of rhodamine 123, a typical substrate of P-glycoprotein (P-gp). The results showed that Zhi Shi significantly decreased the *C*_max_ and AUC_0−*t*_ of tacrolimus by 72.4% and 72.0%, respectively, whereas Zhi Ke did not affect tacrolimus pharmacokinetics. LS 180 cell line study indicated that Zhi Shi increased the efflux activity of P-gp, enabling us to explain the decreased oral bioavailability of tacrolimus caused by Zhi Shi. Hence, we suggest that Zhi Shi be contraindicated for transplant patients treated with tacrolimus to reduce the risk of allograft rejection.

## 1. Introduction

Zhi Ke and Zhi Shi, the dried ripe peels and unripe fruits of *Citrus aurantium, *respectively, are widely used in clinical Chinese medicine. The ripe fruits were harvested in July and the unripe ones were harvested earlier in May-June. Zhi Ke was clinically used in easing pain of epigastrium or abdomen and poor appetite due to stagnation of spleen and stomach [1]. The constituents in Zhi Ke include naringin, naringenin, hesperidin, neohesperidin, *β*-carotene, l-carotene, citraurin, synephrine, N-methyltyramine, auraptenol, aurantiamaric acid, aurantiamarin, and so forth [2, 3]. In contrast, Zhi Shi was often prescribed for gastrointestinal diseases such as food retention, constipation, distention of chest, and epigastrium [1]. The constituents in Zhi Shi include naringin, naringenin, hesperidin, neohesperidin, *β*-carotene, l-carotene, citraurin, synephrine, N-methyltyramine, auraptenol, rhoifolin, lonicerin, poncirin, limonin, 5-*O*-desmethylnobiletin, and so forth [2, 3].

Tacrolimus, a lipophilic macrolide isolated from *Streptomyces tsukubaensis*, is an important immunosuppressant widely used in transplant patients, but with narrow therapeutic window. The half-life of tacrolimus in human is 8.7–11.3 h [4]. The adverse effects of tacrolimus included neurotoxicity, nephrotoxicity, gastrointestinal toxicity, hyperkalaemia, hypertension, and myocardial hypertrophy [5–8]. On the contrary, subtherapeutic level of tacrolimus may result in acute rejection of xenografts [9, 10]. Therefore, whatever significantly affecting the absorption or disposition of tacrolimus is of clinical importance. Tacrolimus is known as a substrate of P-glycoprotein (P-gp), a multidrug efflux transporter, and cytochrome P450 3A4 (CYP3A4) [11–13]. Consequently, any modulator of P-gp or CYP3A4 may alter the pharmacokinetics of tacrolimus.

In recent decades, several clinical studies have found that grapefruit juice and pomelo significantly increased the blood levels of tacrolimus [14–18]. The fruits of *Citrus aurantium *share many common constituents with grapefruit and pomelo, such as naringin, naringenin, hesperidin, and so forth [3]. Therefore, we suspect that Zhi Ke and Zhi Shi may be subject to relevant interaction with tacrolimus as grapefruit juice and pomelo did [15–18]. Our previous study has reported that Zhi Ke significantly increased the oral bioavailability and toxicity of cyclosporine in pigs [19]. Another study found that Zhi Shi significantly decreased the oral bioavailability of cyclosporine in rats [20]. This study in turn investigated the effects of Zhi Ke and Zhi Shi on the pharmacokinetics of tacrolimus in rats. Furthermore, possible mechanism of interaction was explored by using cell line model.

## 2. Methods

### 2.1. Chemicals

 Tacrolimus (Prograf, 5 mg/mL) was purchased from Fujisawa Pharmaceutical Company (Osaka, Japan). The crude drugs of Zhi Ke and Zhi Shi were purchased from a Chinese drugstore in Taichung and identified by microscopic examination. The specimens were deposited at the Institute of Chinese Pharmaceutical Sciences. Dimethyl sulfoxide (DMSO), 3-(4′,5′-dimethylthiazol-2′-yl)-2,5-diphenyltetrazolium bromide (MTT), rhodamine 123, sodium dodecyl sulfate (SDS), Triton X-100, and verapamil were obtained from Sigma (St. Louis, MO, USA). 6,7-Dimethoxycoumarin was purchased from Aldrich (Milwaukee, WI, USA). Dulbecco's Modified Eagle Medium (DMEM), trypsin/EDTA, nonessential amino acid, Hank's Buffered Salt Solution (HBSS), and 4-(2-hydroxyethyl)-1-piperazineethanesulfonic acid (HEPES) were purchased from Invitrogen (Grand Island, NY, USA). IMx kit was supplied by Abbott Laboratories (Abbott Park, IL, USA). Milli-Q plus water (Millipore, Bedford, MA, USA) was used for all preparations.

### 2.2. Instrumentation

 The HPLC apparatus included a pump (LC-10AS, Shimadzu, Japan) and an UV/VIS detector (SPD-10A, Shimadzu, Japan). The RP-18e column (Apollo, 5 *μ*m, 250 × 4.6 mm) was equipped with a guard column (LiChrospher 100, 5 *μ*m). The mobile phase consisted of acetonitrile and water at a ratio of 22 : 78 (v/v). The UV detector was set at 280 nm, and the flow rate was 1.0 mL/min.

### 2.3. Preparation and Characterization of Zhi Ke and Zhi Shi Decoctions

 To prepare decoctions, 25 g of Zhi Ke or Zhi Shi were added to 500 mL of water, and then heated on a gas stove. After boiling, the mixture was heated gently until the volume of decoction was reduced to less than 100 mL. The mixture was filtered while hot, and sufficient hot water was added to make 100 mL. Characterization of the decoctions was carried out through quantitation of naringin and naringenin by HPLC following a previous method with little modification [19]. Briefly, 3.0 mL of decoction was mixed with 7.0 mL of methanol, and centrifuged at 10,000 g for 15 min. A portion of the supernatant was mixed with equal volume of methanol containing 40.0 *μ*g/mL of 6,7-dimethoxycoumarin as internal standard. After filtered through 0.45 *μ*m filter, the sample was subject to HPLC analysis. The remaining decoction was frozen at −30°C for later use.

### 2.4. Animals and Drug Administration

 Male Sprague-Dawley rats were supplied by National Laboratory Animal Center (Taipei, Taiwan) and kept at least 1 week under environmentally controlled conditions with free access to food and water before experiment. After overnight fast, six rats aged 8–12 weaks and weighing 300–400 g were given 1.5 mg/kg of tacrolimus orally with and without decoction of Zhi Ke or Zhi Shi (2 g/kg) in a cross-over design. Drug administration was carried out via gastric gavage, and the decoctions were given 10 min before tacrolimus. Equal volume of water as the decoction was administered as control vehicle. Food was withheld for another 3 h after dosing. One-week was allowed for washout between two treatments. All animal experiments adhered to “The Guidebook for the Care and Use of Laboratory Animals (2002)” (Published by the Chinese Society of Animal Science, Taiwan), and the experimental protocol had been reviewed and approved by the Institutional Animal Care and Use Committee of China Medical University, Taiwan.

### 2.5. Blood Collection and Determination of Tacrolimus Blood Concentration

 Blood samples (0.3 mL) were withdrawn via cardiopuncture at 0, 5, 15, 30, 60, 120, 240, and 480 min after oral dosing of tacrolimus. Blood samples were collected in vacutainer tubes containing EDTA and analyzed within 24 h.

Blood tacrolimus concentration was assayed by a microparticle enzyme immunoassay (MEIA) using IMx kit. All the procedures, calibration curve, and validation followed the working protocols provided by the supplier. The assay was calibrated from 3.0 to 30.0 ng/mL, and the lower limit of detection was 1.5 ng/mL.

### 2.6. Cell Line

 LS 180, the human colon adenocarcinoma cell line, was obtained from the Food Industry Research and Development Institute (Hsinchu, Taiwan). Cells were cultured in DMEM medium supplemented with 10% fetal bovine serum (Biological Industries Ltd., Kibbutz Beit Haemek, Israel), 0.1 mM nonessential amino acid, 100 units/mL of penicillin, 100 *μ*g/mL of streptomycin, and 292 *μ*g/mL of glutamine. Cells were grown at 37°C in a humidified incubator containing 5% CO_2_. The medium was changed every other day, and cells were subcultured when 80% to 90% confluency was reached.

### 2.7. Cell Viability Assay

 The effects of Zhi Shi decoction, verapamil, and DMSO on the viability of LS 180 cells was evaluated by MTT assay [21]. Cells were seeded into a 96-well plate. After overnight incubation, the tested agents were added into the wells and incubated for 72 h, then 10 *μ*L of MTT (5 mg/mL) was added into each well and incubated for additional 4 h. During this period, MTT was reduced to formazan crystal by live cells. Acid-SDS (10%) solution was added to dissolve the purple crystal at the end of incubation, and the optical density was detected at 570 nm by a microplate reader (BioTex, Highland Park, Winooski, VT, USA).

### 2.8. Transport Assay

 The transport assay of rhodamine 123 was modified from a previous method [22]. Briefly, LS 180 cells (1 × 10^5^) were cultured in each well in a 96-well plate. After overnight incubation, the medium was removed and washed three times with ice-cold PBS buffer. Rhodamine 123 in HBSS (10 *μ*M, 100 *μ*L) was added into each well and incubated at 37°C. After 1-h incubation, the supernatants were removed and washed for three times with ice-cold PBS. Then, Zhi Shi decoction, verapamil (as positive control) and DMSO were added to correspondent wells and incubated at 37°C. After 4-h incubation, the medium was removed and the cells were washed three times with ice-cold PBS. Subsequently, 100 *μ*L of 0.1% Triton X-100 was added to lyse the cells and the fluorescence was measured with excitation at 485 nm and emission at 528 nm. To quantitate the content of protein in each well, 10 *μ*L of cell lysate was added to 200 *μ*L of diluted protein assay reagent (Bio-Rad, Hercules, CA, USA) and the optical density was measured at 570 nm. The relative intracellular accumulation of rhodamine 123 was calculated by comparing with that of control.

### 2.9. Data Analysis

 Noncompartment model of WinNonlin (version 1.1, SCI software, Statistical Consulting Inc., Apex, NC, USA) was used for the computation of pharmacokinetic parameters of tacrolimus. The area under the blood concentration-time curve (AUC_0−*t*_) was calculated by the trapezoidal rule to the last point. Pharmacokinetic parameters among various treatment groups were compared using one-way ANOVA with Scheffe's test, taking *P* < .05 as significant.

## 3. Results

**Figure 1:**
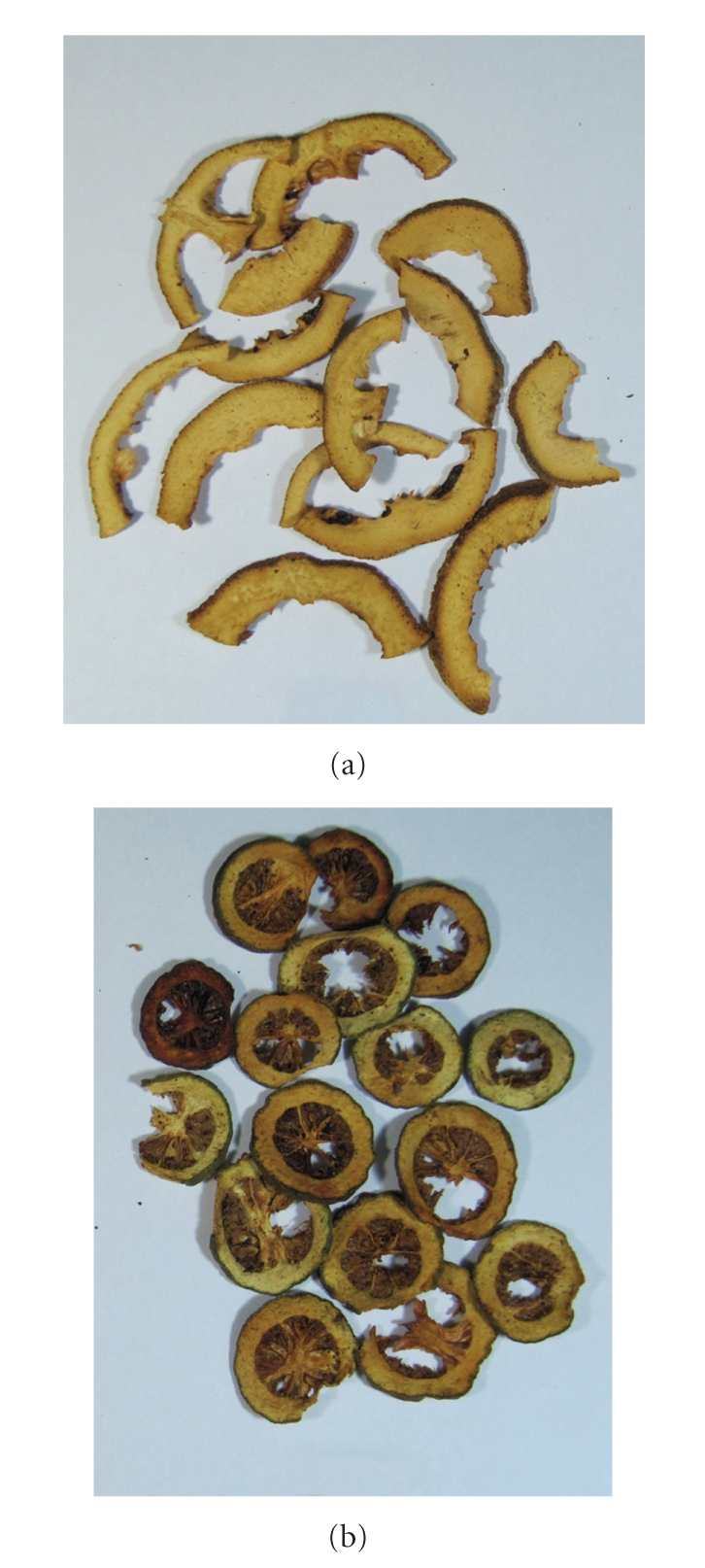
Crude drugs of Zhi Ke (a) and Zhi Shi (b).

Figure 1 is the pictures of crude drugs of Zhi Ke and Zhi Shi. Quantitation results showed that the contents of naringin and naringenin in Zhi Ke decoction were 280. 5 and 5.0 *μ*g/mL, respectively, and in Zhi Shi decoction were 164.3 and 5.2 *μ*g/mL, respectively.

**Figure 2:**
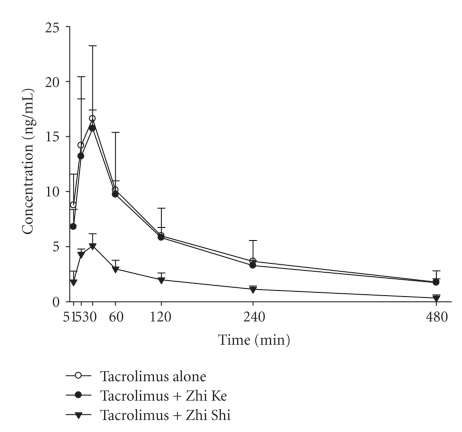
Mean (±S.D.) blood concentration-time profiles of tacrolimus after oral administration of tacrolimus alone (1.5 mg/kg) (°) and coadministrations with 2 g/kg of Zhi Ke (●) and Zhi Shi (*▾*) decoctions.

Figure 2 depicts the mean blood concentration—time profiles of tacrolimus after treatments with tacrolimus alone and coadministered with Zhi Ke and Zhi Shi decoctions in rats. The pharmacokinetic parameters of tacrolimus after various treatments are listed in Table 1. Following coadministration with Zhi Ke, the blood profile of tacrolimus was essentially superposable with that of control. Nonetheless, the blood levels of tacrolimus were markedly decreased since the very early absorption phase upon coadministration with Zhi Shi, which significantly decreased the peak blood concentration (*C*_max_) and area under the concentration curve (AUC_0−*t*_) of tacrolimus by 72.4% and 72.0%, respectively. The time to peak concentration (*T*_max_) and mean residence time (MRT) of tacrolimus were not affected.

**Figure 3:**
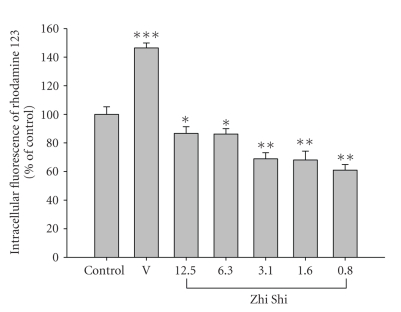
Effects of Zhi Shi (mg/mL) and verapamil (V, 200 *μ*M) on the accumulation of rhodamine 123 in LS 180 cells. Data expressed as mean ± S.D. *P < .05, **P < .01, ***P < .001.

To explore the mechanism of Zhi Shi-tacrolimus interaction, LS 180 cell model was used for the transport assay of rhodamine 123. Through MTT assay, Zhi Shi decoction showed no significant influence on cell viability at the tested concentrations. In transport study, the intracellular accumulation of rhodamine 123 measured after 4-h incubation with tested agents is shown in Figure 3. The positive control verapamil (200 *μ*M) significantly increased the intracellular accumulation of rhodamine 123 by 46.5%. On the contrary, Zhi Shi significantly reduced the intracellular accumulation of rhodamine 123 by 39.0–13.9% at the concentration range of 0.8–12.5 mg/mL.

## 4. Discussions

In this study, a rat model was employed to evaluate the effects of two *Citrus* herbs on tacrolimus pharmacokinetics. The markedly decreased *C*_max_ and AUC_0−*t*_ of tacrolimus caused by coadministration of Zhi Shi indicated that the oral bioavailability of tacrolimus was significantly reduced, whereas Zhi Ke did not alter tacrolimus bioavailability. These results are similar to our previous work reporting the effects of Zhi Ke and Zhi Shi on the pharmacokinetics of cyclosporine, also a substrate of P-gp and CYP 3A4, in rats [20]. For clinical implication, we strongly suggest that concurrent use of Zhi Shi with cyclosporine or tacrolimus should be avoided to reduce the risk of allograft rejection.

Grapefruit juice and pomelo have been well known in that they could elevate the blood levels of cyclosporine and tacrolimus [14, 15, 17, 18, 23, 24]. So far, many constituents have been identified to be the possible causative agents for the interactions between grapefruit juice and western medicines, which were substrates of P-gp or/and CYP3A4 [25]. Among the putative causative constituents, 6′,7′-dihydroxybergamottin and bergamottin, two minor furanocoumarins, have been demonstrated to inhibit enteric CYP3A4 and P-gp [26–32]. However, most of these conclusions were drawn essentially from in vitro studies. Until recently, a human study has pointed out that lack of interaction with felodipine when furanocoumarin-free grapefruit juice was coadministered [33]. As a result, 6′,7′-dihydroxybergamottin and bergamottin can be thought responsible for the inhibition of intestinal CYP3A4 and P-gp resulting from grapefruit juice consumption. Although *Citrus aurantium* belongs to the same genus as grapefruit, our HPLC analysis showed no obergamottin in both Zhi Ke and Zhi Shi decoctions. However, detection of 6′,7′-dihydroxybergamottin in the decoctions was not attempted because of the unavailability of authentic standard. The opposite influences on tacrolimus blood levels between grapefruit juice and Zhi Shi decoction might be in part attributable to the presence of furanocoumarins in grapefruit juice but not in Zhi Shi. 

The origins of Zhi Ke and Zhi Shi are from the fruits of the same plant, but harvested at different seasons. The different influences on tacrolimus pharmacokinetics between coadministrations of Zhi Ke and Zhi Shi might be due to the change of constituents during fruit ripening. Through comparing the concentrations of naringin and naringenin in two decoctions, the involvement of these two flavanones in the interaction can be excluded. On the other hand, the difference of parts used between Zhi Ke and Zhi Shi might be another possible reason to result in different effect. What constituents in Zhi Shi actually resulted in the marked decrease of blood tacrolimus levels is still on the agenda of our future research.

Because the elimination half-lives of tacrolimus between treatments with tacrolimus alone and coadministration with Zhi Shi were not significantly different, we thus propose that the decreased bioavailability of tacrolimus caused by Zhi Shi should primarily occur at the absorption site. Two mechanisms affecting the fate of tacrolimus at absorption site have been identified: pumped out by P-gp in intestine surface at the first stage and, subsequently, the residuals metabolized by CYP3A4 in intestine and liver [11–13]. Therefore, reduced oral absorption of tacrolimus may be associated with the activation of P-gp and/or CYP3A4.

**Figure 4:**
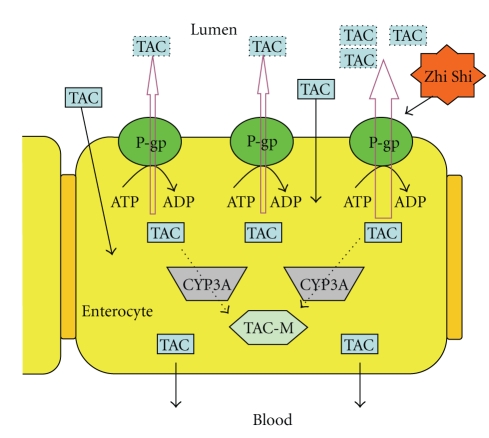
Illustration of the inhibition of Zhi Shi on tacrolimus (TAC) absorption through activating the efflux function of P-gp on the cell membrane of enterocytes. TAC-M: TAC metabolites.

To explore the association of P-gp with the interaction, transport assay of rhodamine 123 was conducted using LS 180 cells. As shown in Figure 3, contrary to verapamil (a positive control as P-gp inhibitor), Zhi Shi decreased the intracellular accumulation of rhodamine 123, indicating that the efflux function of P-gp was activated. The magnitude of P-gp activation was found not proportional to the concentration of Zhi Shi, which might be accounted for by the complex chemical constituents in Zhi Shi decoction. We contemplate that it is likely that some constituents inhibiting P-gp were coexistent in Zhi Shi decoction and counteracted partial P-gp induction effect at high concentration. Based on this in vitro result, the increased P-gp activity might in part explain the decreased blood levels of tacrolimus in rats as illustrated in Figure 4.

 With regard to the effect on CYP3A4, grapefruit juice has been found to decrease intestinal CYP3A4 protein expression [34, 35], which may explain the increased blood levels of tacrolimus [36]. On the contrary, the decreased blood levels of tacrolimus caused by Zhi Shi might stem from induction of CYP3A4 activity. However, an *in vitro* study has revealed that Zhi Shi exerted weak inhibition on CYP3A4 activity in testosterone 6-hydroxylation by human liver microsomes [37], which was apparently not consistent with our *in vivo* evidence. This *in vitro*-*in vivo* discrepancy led us to propose that most constituents in herbal decoctions were hydrophilic and subjected to metabolic transformations in the gut lumen before entering the enterocytes and hepatocytes. How CYP3A4 is involved in herb-drug interaction requires a different approach from drug-drug interaction in western medicines. Therefore, understanding the presystemic metabolism of herbal constituents is essential for the establishment of a rational model more mimicking the biological system.

In conclusion, Zhi Shi significantly decreased the oral bioavailability of tacrolimus in rats, whereas Zhi Ke exerted no influence. We suggest that Zhi Shi be contraindicated for transplant patients treated with tacrolimus to reduce the risk of allograft rejection.

## Figures and Tables

**Table 1:** Pharmacokinetic parameters of tacrolimus in six rats given tacrolimus (1.5 mg/kg) alone and coadministered with 2 g/kg of Zhi Ke and Zhi Shi decoctions.

Parameter		Treatment	
	Tacrolimus alone	Tacrolimus + Zhi Ke	Tacrolimus + Zhi Shi
*T*_max_ (min)	22.5 ± 8.2	27.5 ± 6.1	25.0 ± 7.7
*C*_max_ (ng·mL^−1^)	19.2 ± 4.2^a^	17.7 ± 4.8	5.3 ± 0.8^b^ (−72.4%)
AUC_0−480_ (ng·min·mL^−1^)	2485.4 ± 964.2^a^	2330.5 ± 268.8^a^	696.2 ± 182.5^b^ (−72.0%)
MRT (min)	144.3 ± 15.2	147.6 ± 9.1	127.0 ± 29.9

*T*_max_: time to reach *C*_max_.

*C*_max_: the peak blood concentration.

AUC_0−480_: area under the blood concentration-time curve to 480 min.

MRT: mean residence time.

Data expressed as mean ± S.D. Means in a row without a common superscript differ, *P* < .05.
